# Interfacial Origin of the Magnetisation Suppression of Thin Film Yttrium Iron Garnet

**DOI:** 10.1038/s41598-017-10281-6

**Published:** 2017-09-18

**Authors:** A. Mitra, O. Cespedes, Q. Ramasse, M. Ali, S. Marmion, M. Ward, R. M. D. Brydson, C. J. Kinane, J. F. K. Cooper, S. Langridge, B. J. Hickey

**Affiliations:** 1 School of Physics and Astronomy, University of Leeds, Leeds, LS2 9JT UK; 2 School of Chemical and Process Engineering, University of Leeds, Leeds, LS2 9JT UK; 3 SuperSTEM Laboratory, SciTech Daresbury Campus, Keckwick Lane, Daresbury, WA4 4AD UK; 4 Rutherford Appleton Laboratory, ISIS, Science and Technology Facilities Council, Didcot, OX11 0QX Oxon UK

## Abstract

Yttrium iron garnet has a very high Verdet constant, is transparent in the infrared and is an insulating ferrimagnet leading to its use in optical and magneto-optical applications. Its high Q-factor has been exploited to make resonators and filters in microwave devices, but it also has the lowest magnetic damping of any known material. In this article we describe the structural and magnetic properties of single crystal thin-film YIG where the temperature dependence of the magnetisation reveals a decrease in the low temperature region. In order to understand this complex material we bring a large number of structural and magnetic techniques to bear on the same samples. Through a comprehensive analysis we show that at the substrate -YIG interface, an interdiffusion zone of only 4–6 nm exists. Due to the interdiffusion of Y from the YIG and Gd from the substrate, an addition magnetic layer is formed at the interface whose properties are crucially important in samples with a thickness of YIG less than 200 nm.

## Introduction

Recent research^1–3^ in spintronics has focused on magnetic insulators to develop efficient magnetic-based devices using spin currents. YIG is a ferrimagnetic insulator with a band gap of 2.58 eV^4^, and attracts considerable interest due to its extremely small magnetisation damping:^5^ *α* ≈ 3 × 10^−5^. As YIG is an insulator, it is free from parasitic heating effects due to charge currents, and is ideal for use in spin pumping, spin transfer torque and spin Hall magnetoresistance applications. In addition, since the spin wave decay length in YIG is several centimeters^6^, spin currents can propagate over long distances which has given impetus to the burgeoning field of *magnonics*.

Traditionally YIG has been grown by several techniques with liquid phase epitaxy (LPE)^7–9^ being the most successful at obtaining the highest quality, but in rather thick films (~microns). There are several reports on the deposition of nm-thick YIG films by pulsed laser deposition (PLD)^10^ or a UHV variant^11^. However, the best results for PLD samples seems to be from Hauser *et al*. where damping as low as 7 × 10^−5^ for a 20 nm film has been reported^12^. RF sputtering followed by either *in situ* annealing^13,14^ or post-growth annealing^3,15,16^ has attracted considerable interest. On the other hand, *off-axis* sputtering^15,17^ has been reported to produce highly crystalline material with reasonable magnetic properties measured at room temperature.

Gadolinium Gallium Garnet (Gd_3_.Ga_5_O_12_) is the substrate of choice for growing YIG because in the (111) orientation there is a lattice mismatch of only 0.06%. All of the growth techniques for YIG involve the use of high temperatures (>700 °C) either during growth or in post-growth annealing. We are especially interested in the relationship between the structural and magnetic properties of YIG where we believe small changes at the interface can have significant effects on the more desirable properties of YIG. We have adopted a multi-technique approach here and note that measuring the magnetic moment of YIG at low temperatures is quite challenging as GGG is a paramagnet that easily swamps the magnetic signal from YIG. Yet, the magnetic properties are particularly important, for example, in spin transfer torque applications as the demand for thin film YIG in the region of 10–20 nm increases. Here we report on high quality, smooth, nm-thick YIG films on GGG grown by on-axis RF sputtering.

## Results

**Figure 1:**
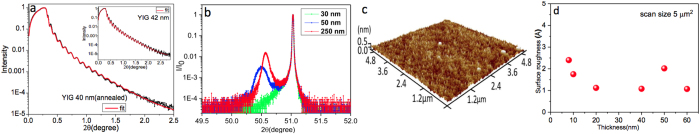
Panel (a) shows the X-ray reflectivity (Cu K_*α*_, *λ* = 1.54 Å radiation) of a typical YIG film where the inset shows the sample before annealing. The points are the measured data and the line is a best fit whose parameters are cited in the table. A very good fit is obtained to the unnealed sample with a single layer of YIG. However, a good fit could only be obtained for the annealed sample by incorporating an interface region instead of a sharp GGG/YIG interface. The YIG has a (111) crystalline orientation and films develop a diffraction peak (panel b, the two peaks near 50.5°) as a function of thickness that corresponds to a lattice constant of 12.496 ± 0.002 Å compared to 12.376 Å for bulk YIG. The peak at 51.05° corresponds to the GGG substrate with the lattice constant of 12.383 Å. Average surface roughness was measured by atomic force microscopy (AFM) over an area of 5 × 5 microns (panel c). The films’ surface morphology is depicted in panel (d) where it can be seen that the surfaces are smooth with an RMS roughness of about 1–3 Å.

Figure 1a shows an example of the X-ray reflectivity (XRR) of a typical YIG sample before and after annealing (see Table 1 for parameters). For the unnealed sample the electron densities of both the substrate and YIG are about 5% less than the bulk. After annealing the total thickness is diminished but the important point is that we cannot obtain the best fit for these data without including an additional layer at the interface. The indication that there may be diffusion at the interface is that although a good fit was obtained using only a YIG layer (goodness of fit (GOF) of 0.05), the fitted density of the GGG was only 91% that of bulk GGG. A much better fit is obtained by using a trilayer system of GGG/~50–60 Å of Gd_3_Fe_5_O_12_/YIG where the interface layer has a density that is reduced by about 13%. In the light of additional structural information (see TEM results below), we now model this interface as a mixed layer of YIG and Gd (YIG1 and Gd in Table 1). This illustrates that we are not relying on a particular crystal structure (GdIG, for example) to explain the results. The interface layer is modelled by allowing the total thickness to approach that of the dead layer (6 nm) found in magnetometry and significant roughness and grading in the layer. The roughness in this fit is modelled as a Gaussian with the full width at half maximum representing the standard deviation of an effective roughness. The roughness is that produced by terraces or steps, for example, whereas grading is represents interdiffusion. However, since we are only in the specular regime, it is not possible to distinguish between a chemically graded interface and physical roughness^18^. Nevertheless, the parameters returned by the fit for this layer are as we expected - roughness and grading that is nearly equal to the thickness of the layer and much reduced densities. In this way we can represent a somewhat disordered interface layer. The roughness at the other interfaces are reasonable and given that the x-rays illuminate the entire sample, 4 Å roughness on the YIG surface agrees quite well with the RMS roughness of the atomic force microscopy (AFM) results. The thickness of the interface region varies from 5–6 nm between samples. The GOF for this fit is 0.04 and importantly, the densities for the YIG and GGG are within 1% of the bulk values giving us confidence that the top layer is stoichiometrically correct.

**Table 1:** Parameters used in fitting the x-ray reflectivity shown in 1a. The densities are expressed as a per cent of the bulk layer.

**Unnealed**				
**Layer**	**Thickness (Å)**	**Density (%)**	**Roughness (Å)**	**Grading (Å)**
YIG	422	96.4	4.6	—
GGG	∞	96.9	6.0	—
**Annealed**				
YIG	347	99.3	4.3	0.0
YIG1	29	93.2	14	2.3
Gd	18	89.1	7	19
GGG	∞	99.9	8.8	0.0

Figure 1b presents the X-ray diffraction (XRD) data for three samples of YIG with thicknesses 30, 50 and 250 nm. These data show the evolution of a peak that, for the thickest sample, has a lattice constant of 12.496 ± 0.002 Å. This shows that the YIG film is close to the (111) crystalline orientation of the GGG substrate with a lattice constant of 12.383 Å for the (444) planes (seen at 51.05°). The diffraction peak width is 0.0120 ± 0.0001 degrees confirming the high quality of the films. To assess the roughness of the YIG surface, AFM has been performed in tapping mode over a range of 5 microns. As can be seen from the image in Fig. 1(c), the surface appears smooth over this range and the rms roughness of a series of films in the 6–70 nm thickness range (panel d) have surface roughness of 1–3 Å.

**Figure 2:**
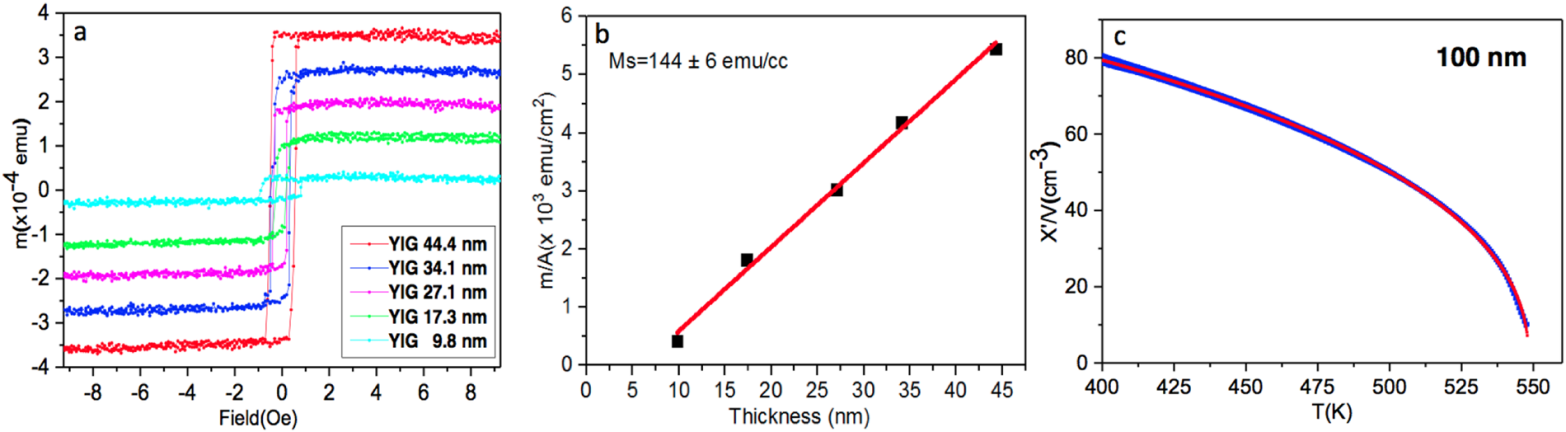
Panel (a) shows the data for M(H) at room temperature for a range of thickness. In panel (b) we plot the magnetic moment per area as a function of thickness which indicates that there is a *dead layer* of about 6 nm and the linear relation indicates there is a single value of the magnetisation (144 ± 6 emu/cc). The Curie temperature is determined by a fit to the Bloch T^3/2^ law (panel c) and gives a value close to the bulk value of 559 K.

The magnetic properties of YIG films were studied at 295 K using a vibrating sample magnetometer (VSM) by applying an in-plane magnetic field. Figure 2(a) shows the hysteresis loops for different YIG film thicknesses ranging from 10–50 nm. The data shows that the coercivity is similar across this range of samples with a value of 0.30 ± 0.05 Oe. In order to determine the magnetisation of our samples we have plotted the magnetic moment per unit area a function of thickness. The results are shown in Fig. 2(b). From these data it is clear that the relationship is linear and that the data does not extrapolate to zero for zero thickness. Thus there is a single value of the magnetisation that describes these data (144 ± 6 emc/cc) which compares well with the bulk value of 140 emu/cc^19^. In addition, at room temperature, there is a magnetic *dead layer* of about ~6 nm. As is common in magnetic thin films, dead layers are usually found at the sample/substrate interface and in this case this thickness corresponds remarkably well to the diffusion region identified by the reflectivity and the structural analysis (see below). The interpretation thus far is that Gd and Y have interdiffused into the interface region but at room temperature it is paramagnetic because 295 K is above the Curie temperature of the Gd-doped region. Panel (c) of Fig. 2, shows the Curie temperature of a 100 nm sample fitted with a Bloch T^3/2^ law illustrating that the value of ~550 K is close to the bulk value of 559 K.

**Figure 3:**
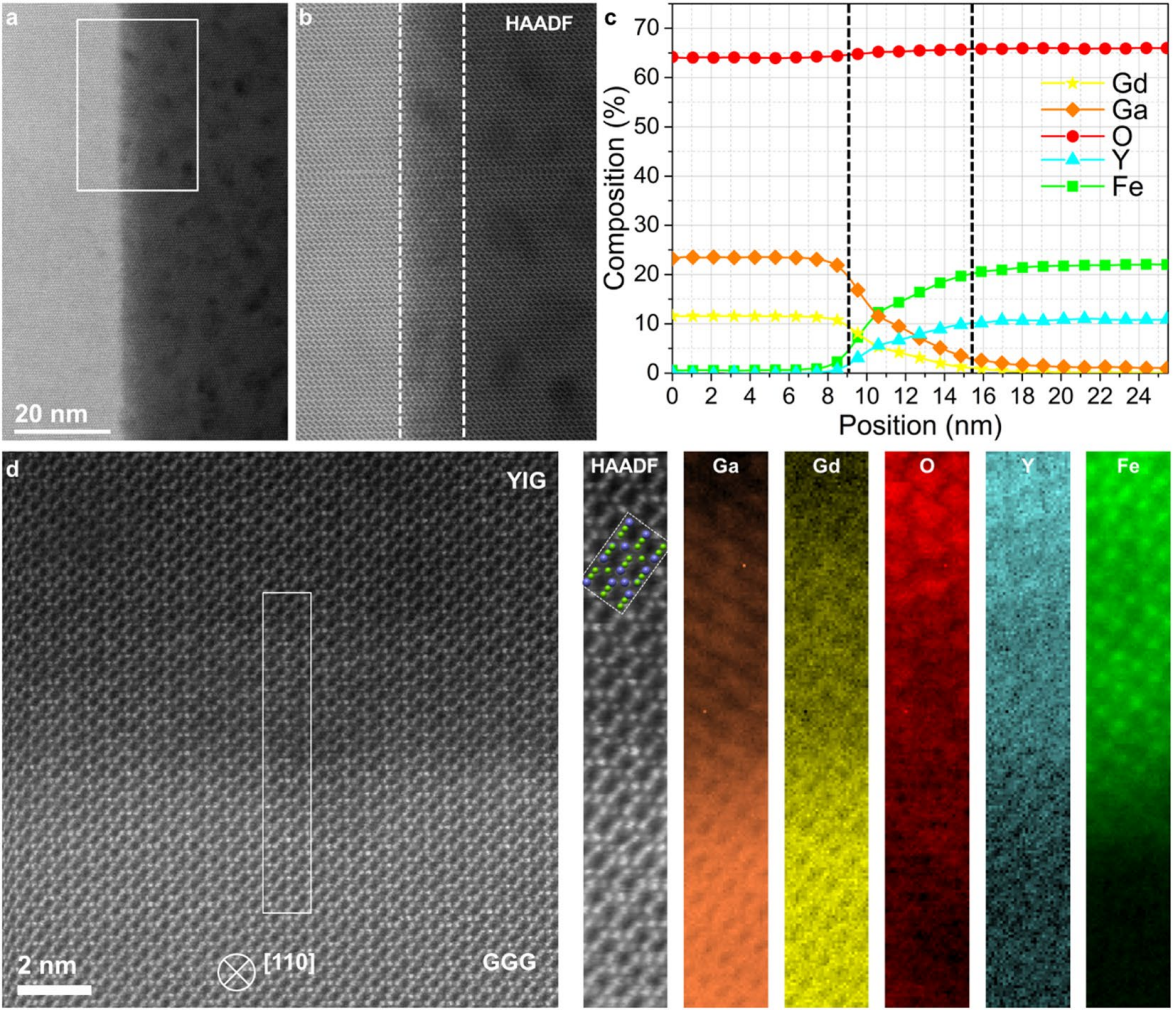
Analytical STEM characterisation of the YIG/GGG interface. (**a**) HAADF survey image of the interface (GGG:left, YIG:right). A white box indicates the region over which the EELS spectrum imaging was carried out, yielding compositional profiles (obtained using using tabulated Hartree-Slater cross-sections) averaged across the interface: (**c**). The HAADF intensity recorded simultaneously with the EELS maps is shown in (**b**) with white dotted lines indicating as a guide to the eye a ∼6 nm wide region over which some interdiffusion of Gd, Y, Ga, and Fe is observed (corresponding to the region marked with dotted lines on the profiles in (**c**)). Panel (d) shows a higher magnification EELS analysis of the interface (rotated 90 degrees to (**a**)) in the region indicated by a white box on the HAADF survey image. Maps for Ga, Gd, O, Y, and Fe are presented, along with the simultaneously acquired HAADF intensity, over which a ball model of YIG in [110] orientation is overlaid (green balls represent Fe, blue balls represent Y, and oxygen is not represented for clarity).

In order to obtain direct confirmation of interdiffusion, we carried out an atomic-scale investigation of the GGG/YIG interface using aberration-corrected scanning transmission electron microscopy (STEM) (see the methods section for technical details). High-angle annular-dark-field (HAADF) images of the interface observed along the [110] zone axis reveal a gradual transition of the intensity from the GGG substrate to the film: a representative example is shown in Fig. 3a, with identical observations along the entire interface. Given the sensitivity of this imaging mode to the average atomic number, Z, of the material (the HAADF contrast scales to a good approximation as Z^1.7^)^20^, this alone suggests a chemically-diffuse, rather than sharp, interface, in good agreement with the XRR measurements. Electron energy loss spectroscopy (EELS) provides further proof of the interdiffusion of the various cations across the interface: chemical maps of Ga, Gd, O, Y and Fe were recorded across the interface region delimited by the white rectangle in Fig. 3a. Averaged composition profiles obtained by integrating these maps across the interface show a 6.5 nm wide region of mixed chemical composition, while the compositions on either side of this region correspond (within the measurement accuracy: see methods section) to the expected bulk values for YIG and GGG: Fig. 3b. This interdiffusion layer is delimited, as a guide to the eye, by dotted lines on Fig. 3b and on the HAADF image acquired simultaneously with the EELS (Fig. 3c). The shape of the EELS element intensity profiles shown in Fig. 3c are consistent with diffusion of Gd and Ga from the GGG substrate into the deposited YIG layer. For Yttrium and Iron the element intensity profile is inverted, which most likely infers diffusion of vacancies from within the YIG layer (presumably arising as a result of deposition) to the original GGG/YIG boundary (which acts as a vacancy sink) and hence diffusion of Y and Fe away from the boundary. This would then ultimately result in a Gd- and Ga-doped YIG interlayer some 6 nm thick. Images and chemical maps obtained at higher spatial sampling provide an atomic-scale picture of the interface, although due to tight packing in this orientation, it is difficult to confirm the exact lattice position of the interdiffused cations: Fig. 3d. Nevertheless, it is clear from these results that the extent and chemical nature of the interdiffusion is in remarkable agreement with the conclusions drawn from the other techniques. It is known that Gd and Y are diffusion pairs in the GGG/YIG system^21^ diffusing with similar coefficients through the (c) sublattice. An interdiffusion distance of width of 6 nm implies a diffusion lengthscale of ∼3 nm either side of the original boundary. From the annealing conditions (2 hours at 850 °C), we estimate a diffusion coefficient of ∼1.25 × 10^−17^ cm^2^ s^−1^. This compares favourably with an extrapolated diffusion coefficient for Y in YIG at 850 °C of between 10^−17^ and 10^−18^ cm^2^ s^−1^ (from Fig. 8 in ref.^22^). We note that Gallagher *et al*.^23^ show a STEM/EDX profile across a similar YIG/GGG interface which exhibited an interfacial transition regions of ca. 5 nm (for Gd and Fe). They attributed this to delocalisation of the X-ray emission due to probe broadening and inelastic delocalisation rather than elemental interdiffusion; however, this delocalisation appeared to vary between different elements. We believe our current STEM/EELS results, which were taken from a large number of separate regions along the interface, do not suffer such problems with delocalisation and can be attributed to chemical intermixing matching the structural data from the other techniques.

**Figure 4:**
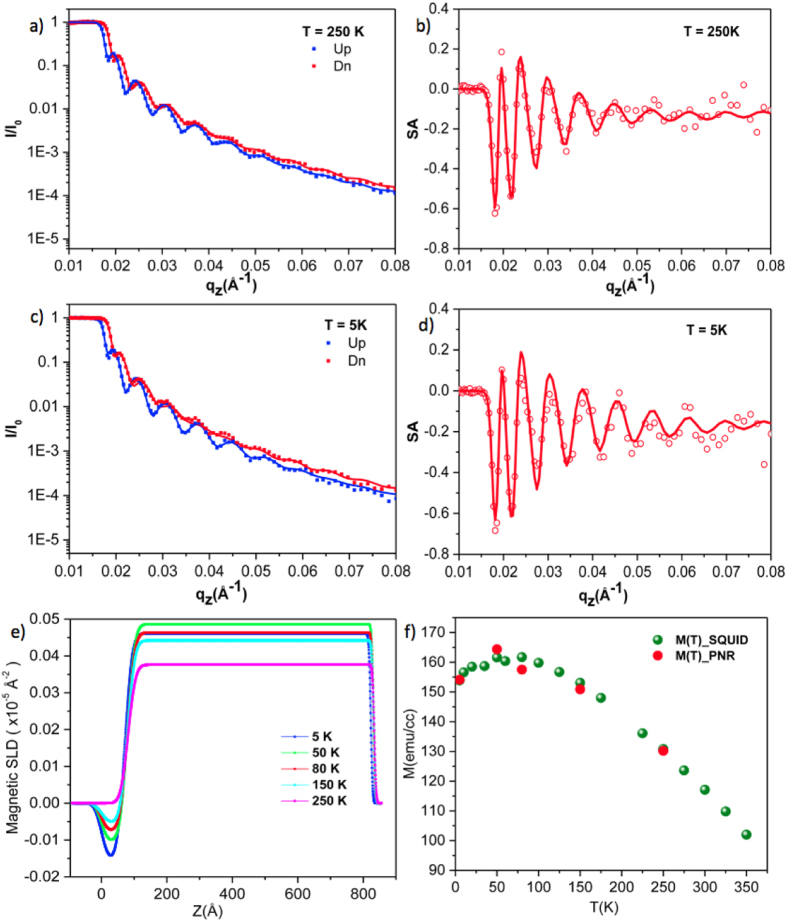
Spin-polarised neutron reflectivity data for an 80nm-thick YIG film at 250 K (panel (a)) and 5 K (panel (c)). Panels (b) and (d) show the extracted spin asymmetry data along with their fits. In panel (e) we show the scattering length density (SLD) as a function of distance through the sample (z = 0 is the GGG/YIG interface) and temperature. Clearly visible is the increase in the Gd moment ordering antiparallel to the YIG moment (between 0 and 50 Å) as the temperature is reduced. Panel (f) shows the temperature dependence of the magnetisation for the same 80 nm sample measured by SQUID magnetometry. The points overlaid are the integrated SLD which shows a convincing correlation between the two measurements.

It would seem that the room temperature magnetic properties of YIG do not reveal any influence of the Gd diffusion but, as a function of temperature, the magnetic ordering of the Gd-diffused layer is immediately evident in the magnetisation. To better understand the magnetic behaviour in this complex material we require a depth-resolved technique. Polarised neutron reflectivity (PNR) is ideally suited and measurements were performed on the Polref beamline at the ISIS neutron source of the Rutherford Laboratory where there is the availability of a range of temperatures and applied magnetic fields. We measured the temperature dependence of the magnetisation, M(T), from 385 K down to 1.8 K using a SQUID-VSM in an applied field of 30 mT and have analysed these in combination with the PNR measurements as a function of temperature on the same samples. Figure 4(a) and (c) show the temperature dependent spin-polarised neutron reflectivity data and their fits for an 80nm-thick YIG sample. Derived from these data is the spin asymmetry (SA), shown in panels (b) and (d), which is given by:

1 $$SA=\frac{{I}_{\uparrow }-{I}_{\downarrow }}{{I}_{\uparrow }+{I}_{\downarrow }}$$

The fits for the reflectivity and SA are obtained by using Gen-X^24^ and a two layer model for YIG and Gd-doped YIG. The PNR model has a rough substrate (∼1.2 nm) and then a layer of 6–7 nm for the interdiffusion region. Recall we see the average scattering length density so we cannot make the elemental distinction. However Gd is one of the few elements with a significant neutron absorption cross-section which makes us more sensitive to it. The absorption is included in the neutron model and has to be there for the model to fit the data reliably. From the model we obtain the scattering length densities (see Methods section) plotted as a function of distance and temperature in panel (e) of Fig. 4. The z-axis represents distance through the vertical direction of the sample where z = 0 indicates the GGG/YIG interface. At 250 K the SLD near z = 0 indicates a region that is paramagnetic but as the sample is cooled, this region becomes magnetic and orders anti-parallel to the rest of the YIG: indicated by a negative SLD. The total thickness is given by the Kiessig fringes and the model returns a thickness of ∼6 nm for the Gd-diffusion region which agrees well with the X-ray, the superSTEM and the room temperature magnetometry. The integrated SLD is proportional to the magnetisation of the sample and these data are plotted on the independently measured M(T) in 3(f). It is obvious that the data agree well. The moment in YIG is due to antiferromagnetic superexchange mediated by the O^2−^ between the Fe^3+^ ions on the A and D sites. This is the strongest of the interactions, which is why Gd-doping does not change T_*c*_. Gd substitutes for Y on the C sites and orders antiparallel to the net moment of the A + D sites. This explains the observed PNR results. In Gd-doped compounds where the concentration of Gd exceeds 24%, there is a compensation temperature where the total moment passes through zero and interestingly, the Gd-YIG system rotates coherently as the compensation temperature is passed^19^.

Informed by the PNR results, we developed a mean field model for the YIG system based on two layers where:

2 $$M(T)={M}_{y}B({J}_{y},{z}_{y})+{M}_{g}B({J}_{g},{z}_{g})$$

where *B* is the Brillouin function, *M*_*y*_ and *M*_*g*_ are the saturation magnetisations of the YIG and Gd-YIG layers respectively.

3 $${z}_{y}=\frac{g{\mu }_{B}{H}_{y}{J}_{y}}{{k}_{B}T}(\frac{t-{t}_{g}}{t}){M}_{m}(T)$$

and

4 $${z}_{g}=\frac{g{\mu }_{B}{H}_{g}{J}_{g}}{{k}_{B}T}(\frac{{t}_{g}}{t}){M}_{m}(T)$$

where *t* is the thickness of the total layer, *t*_*g*_ is the thickness of the Gd-layer, taken to be 6 nm for all samples, *H*_*y*_ and *H*_*g*_ are the Weiss fields for YIG and the Gd-layer and are fitting parameters. *M*_*m*_(*T*) is the measured value of the magnetisation. The spin quantum numbers for the two layers were taken to be: *J*_*y*_ = 5/2 and *J*_*g*_ = 7/2.

**Figure 5:**
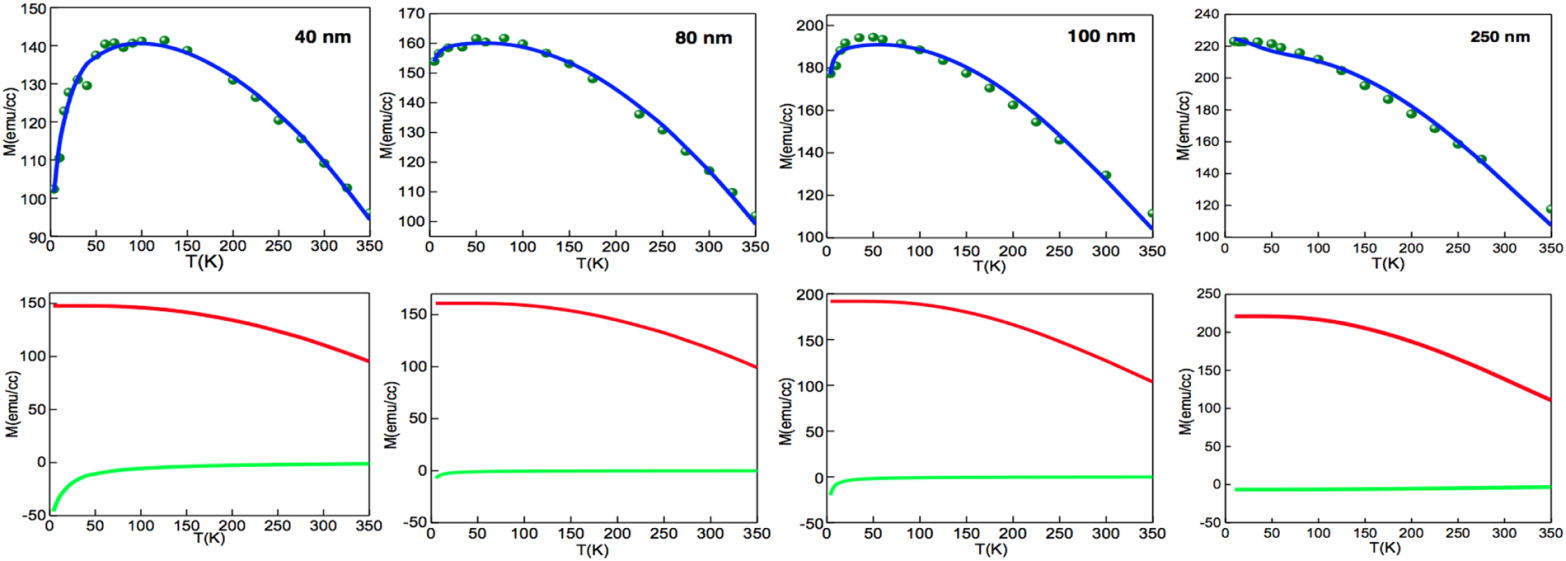
Temperature dependence of magnetisation for a series of film thicknesses. The top panels show the M(T) data where the smooth curve is a fit to the data using a mean-field approach. The model uses two magnetic layers: YIG and a Gd-rich diffused layer which, below about 100 K, is magnetic and orders antiparallel to the YIG, The lower panels show the magnetisation for the individual layers in the model: YIG in red and the Gd-layer in green. The negative values for the Gd-layer indicates it is antiparallel to the YIG.

Figure 5 shows the temperature dependence of the saturation magnetisation where below 100 K there is the decrease in M(T) where the extent of the decrease depends on the thickness of the YIG. In the upper panels are the M(T) measurements for various thicknesses of the samples and the solid line represents the fit using the two-layer mean field model. The lower panels show the two individual layer magnetisations where a negative sign indicates an antiparallel moment. The fits return values of the saturation magnetisation for YIG that are close to the accepted value. The behaviour of the Gd-layer varies slightly across the samples, since we have taken a fixed thickness of 6 nm for this layer, the difference is due to the Weiss field fitting parameter. We note that it is very difficult for the fitting routine to find an acceptable Gd-layer result for the thickest samples as there is no decrease in M(T) - without this, a single layer fit will suffice. We have kept the thickness of the Gd-layer constant as that is what is found in the other techniques but we don’t know the concentration of the elements in the diffusion layer. We have assumed from the profile of the diffusion elements (Fig. 2c) that vacancies might be implicated. It is possible that the concentration of vacancies after annealing depends on the thickness of the YIG layer such that the thinner samples have a higher concentration.

## Discussion

It is interesting to ask how the Gd-layer may affect results: an obvious effect could be line broadening in FMR experiments - these can be due to two-magnon scattering^25^ but might also be due to inhomogeneities in the sample^26^. It may be that the Gd appears as impurities that could be seen, for example, in low temperature increases in the FMR linewidth^27,28^ as was recently evoked as an explanation for low temperature damping^29^. In phenomena that rely on the magnon propagation length^30^, and where the YIG films are thin, then we expect the Gd-layer, which will be paramagnetic at room temperature and will gradually align antiparallel as the sample is cooled, will have to be considered. On the other hand those effects that rely only on the orientation of the magnetisation, such as the spin Hall magnetoresistance will be unaffected^31^. It is difficult to say how noticable Gd-Y interdiffusion will be, one has to look for it. In earlier studies of diffusion in bulk samples, the annealing temperatures were much higher, 1000–1600 °C^22^. There is some indication that Gd diffusion is commonplace in thin film YIG grown on GGG, for example in Fig. S2 of the supplementary material^29^ and the magnetisation as a function of thickness reported in Table 1 ^25^ extrapolates to a dead layer of 6.2 nm. It’s possible to limit the diffusion by lowering the temperature, but often this is counterbalanced by an increase in the time and the need to crystallise the structure. There is the possibility of using an annealing step for the GGG before growth or introducing a diffusion barrier but these ideas require further study.  In conclusion this work demonstrates the growth of high quality nm-thick YIG by sputtering and the influence of high temperatures on the structural and magnetic properties. Through a comprehensive study of the morphology, crystallinity, the chemical nature of the YIG/GGG interface, layer-sensitive PNR and SQUID magnetometry we have revealed the nature of this very promising material. We have found that the 6 nm Gd-doped region of the YIG is paramagnetic at room temperature and orders antiparallel to YIG at lower temperatures. This behaviour should be taken into account in, for example, spin pumping and spin torque experiments. The demand for thin film YIG is set to increase as the field of magnonics and insulator-based spintronics develops.

## Methods

YIG films were grown on 0.5 mm thick single-crystalline cubic 〈111〉 GGG substrates by RF magnetron sputtering following an established recipe^31^ using a chamber with a base pressure of 2 × 10^−8^ Torr. YIG was sputtered from a single stoichiometric target using a power of 54 W at 13.56 MHz balanced with variable inductors and capacitors so that the reflected power was always much less than 1 W. During the deposition the argon and oxygen flow rates were 22.4 sccm and 1.2 sccm respectively to maintain the proportion of O_2_ to Ar at 5% at a pressure of 2.4 m Torr. The as-deposited YIG films were amorphous and non-magnetic therefore after deposition the films were annealed at 850 °C for two hours under open air conditions in a tube furnace. The heating and cooling cycles are run at a rate of 7 °C per minute to avoid introducing strain into the film.

Scanning transmission electron microscopy (STEM) and electron energy loss spectroscopy were carried out on a Nion UltraSTEM100 instrument operated at 100 kV acceleration voltage. The optics were adjusted to form a 0.9 Å (full-width at half-maximum) electron probe with a convergence semi-angle of 31 mrad and a beam current of 50 pA. The high-angle annular dark field detector semi-angular range was calibrated as 82–185 mrad. The microscope is equipped with an Enfina electron energy loss spectrometer. EEL spectra were recorded through 36 mrad semi-angle collection aperture, and the dispersion was adjusted to record all edges of interest simultaneously, resulting in an effective resolution (limited by the camera point spread function) of 3.2 eV. After de-noising by principal components analysis^32^ the background was removed by fitting a power law over a region immediately in front of the core loss edges. The signal was then integrated over a 60 eV window above the edge onsets. The contrast in each of the colour maps is normalised to [0, 1] for simplicity (and therefore do not reflect composition, merely the relative spatial distribution of a given element). Because of edge overlaps, the faint M_2,3_ edge of Gd was used for mapping although the signal was good enough in this case to produce a clear map. Composition profiles were estimated (and averaged across the interface) from the EELS maps using tabulated Hartree-Slater cross-sections. These are known to be inaccurate for most of the edges in these compounds. The compositions should therefore only be seen as indicative of trends and the uncertainty on these numbers will be of the order of 10%. The results are almost certainly influenced by the fact that we are on-zone and channelling is boosting the signal for some elements compared to others. By tilting the sample far off-zone, the concentrations would have been closer to stoichiometric values.

Magnetic characterisation was performed in the temperature range 1.8 K to 350 K in a vibrating sample superconducting quantum interference device (SQUID) magnetometer:MPMS3 by Quantum Design. The magnetic field was applied parallel to our samples. Hysteresis loops were recorded at the corresponding temperatures. The paramagnetic slope of the GGG substrate was subtracted from each loop to extract the magnetic moment at each temperature. X-ray data was collected on a Bruker D-8 Discover diffractometer using Cu K_*α*_, *λ* = 1.54 Å radiation. The samples were taken to the polarised neutron reflectometer, POLREF, at the ISIS neutron spallation source, Rutherford Appleton Laboratory for our temperature dependent polarised neutron reflectivity (PNR) measurements.The samples were saturated using a GMW electromagnet in a field of 300 mT, far in excess of the necessary field to saturate the sample. For neutron reflectivity data, a neutron spin flipper was used to record both the up- and down-spin neutrons. The samples were then field-cooled in a standard Oxford Instruments flow cryostat at the saturating field to 250 K, where the initial PNR measurement was performed. Measurements were then done in the following order at 5 K, 80 K, 50 K and 150 K, without changing the applied field at any point, in an effort to make the best use of the counting time available. The samples had dimensions of 20 mm × 20 mm on a 0.5 mm thick GGG substrate. The areas under the SLD curves were integrated (*β*_*SLD*_) and converted to a magnetisation (emu/cc) using *m* = *β*_*SLD*_/*nc* where *m* is the magnetisation, *n* is the atomic number density, and *c* = 2.853 × 10^−9^ cm^3^/emu^33^.
